# The Costs of Creatinine Testing in the Context of a HIV Pre-Exposure Prophylaxis Demonstration Project in Eswatini

**DOI:** 10.1007/s10461-021-03432-4

**Published:** 2021-08-18

**Authors:** Stefan Kohler, Rumbidzai Ndungwani, Mark Burgert, Dumile Sibandze, Sindy Matse, Anita Hettema

**Affiliations:** 1 Faculty of Medicine and University Hospital, Heidelberg Institute of Global Health, Heidelberg University, Im Neuenheimer Feld 130.3, 69120 Heidelberg, Germany; 2 Institute of Social Medicine, Epidemiology and Health Economics, Charité–Universitätsmedizin Berlin, corporate member of Freie Universität Berlin and Humboldt-Universität zu Berlin, Berlin, Germany; 3 Clinton Health Access Initiative, Mbabane, Eswatini; 4 Eswatini Ministry of Health, Mbabane, Eswatini

**Keywords:** Blood test, Costs, Creatinine level, Kidney function test, Laboratory costs, Micro-costing study, Sub-Saharan Africa, Análisis de sangre, Costes, Nivel de creatinina, Prueba de función renal, Costes de laboratorio, Estudio detallado de los costes, África subsahariana

## Abstract

HIV treatment and prevention as well as other chronic disease care can require regular kidney function assessment based on a creatinine test. To assess the costs of creatinine testing in a public health care system, we conducted activity-based costing during a HIV pre-exposure prophylaxis (PrEP) demonstration project in the Hhohho region of Eswatini. Resource use was assessed by a laboratory technician and valued with government procurement prices, public sector salaries, and own cost estimates. Obtaining a blood sample in a clinic and performing a creatinine test in a high-throughput referral laboratory (> 660,000 blood tests, including > 120,000 creatinine tests, in 2018) were estimated to have cost, on average, $1.98 in 2018. Per test, $1.95 were variable costs ($1.38 personnel, ¢39 consumables, and ¢18 other costs) and ¢2.6 were allocated semi-fixed costs (¢1.1 laboratory equipment, ¢0.85 other, ¢0.45 consumables, and ¢1.3 personnel costs). Simulating different utilization of the laboratory indicated that semi-fixed costs of the laboratory (e.g., equipment purchase or daily calibration of the chemistry analyzer) contributed less than variable costs (e.g., per-test personnel time and test reagents) to the average creatinine test cost when certain minimum test numbers can be maintained. Our findings suggest, first, lower creatinine testing costs than previously used in cost and cost-effectiveness analyses of HIV services and, second, that investment in laboratory equipment imposed a relatively small additional cost on each performed test in the high-throughput referral laboratory.

## Resumen

El tratamiento y la prevención del VIH, así como el cuidado de otras enfermedades crónicas, pueden requerir una evaluación periódica de la función renal basada en una prueba de creatinina. Para evaluar los costes de las pruebas de creatinina en un sistema de atención sanitaria público, realizamos un cálculo de costes basado en actividades durante un proyecto de demostración de profilaxis preexposición al VIH (PrEP) en la región de Hhohho de Eswatini. El uso de los recursos fue evaluado por un técnico de laboratorio y valorado con los precios de adquisición del gobierno, los salarios del sector público y las estimaciones de costes propias. La obtención de una muestra de sangre en una clínica y la realización de una prueba de creatinina en un laboratorio de referencia de alto rendimiento (> 660.000 pruebas de sangre, incluidas > 120.000 pruebas de creatinina, en 2018) se estimó que habían costado, en promedio, $1,98 en 2018. Por prueba, $1,95 eran costes variables ($1,38 de personal, ¢39 de consumibles y ¢18 de otros costes) y ¢2,6 eran costes semifijos asignados (¢1,1 de equipamiento de laboratorio, ¢0,85 de otros, ¢0,45 de consumibles y ¢1,3 de personal). La simulación de utilización diferente del laboratorio indicó que los costes semifijos del laboratorio (por ejemplo, la compra de equipos o la calibración diaria del analizador químico) contribuyeron menos que los costes variables (por ejemplo, el tiempo del personal por prueba y los reactivos de la prueba) al coste medio de la prueba de creatinina cuando se pueden mantener ciertos números mínimos de pruebas. Nuestros resultados sugieren, en primer lugar, que los costes de las pruebas de creatinina son inferiores a los utilizados anteriormente en los análisis de coste y costo-efectividad de los servicios de VIH y, en segundo lugar, que la inversión en equipos de laboratorio supuso un coste adicional relativamente pequeño en cada prueba realizada en el laboratorio de referencia de alto rendimiento.

## Introduction

HIV treatment and prevention as well as other chronic disease care can require regular kidney function assessment based on a creatinine test [1–3]. The drug tenofovir disoproxil fumarate (TDF), for instance, is widely used in antiretroviral therapy and pre-exposure prophylaxis (PrEP) of HIV. As TDF has been associated with renal impairment, the World Health Organization advises, if feasible, to monitor the serum creatinine level of people with a high risk of kidney disease receiving TDF [4].

In Eswatini, a 2017–19 demonstration project introduced the provision of PrEP to everyone at a high risk of HIV infection through public primary care clinics [5, 6]. PrEP provision through public primary care clinics has been scaled-up nationwide in Eswatini since the demonstration project [7, 8]. The introduction and expansion of PrEP has increased the demand for creatinine testing in the public health care system. Increased demand for creatinine testing within HIV services due to PrEP expansion could continue until the prevalence of HIV and, thus, the number of people whose kidney function is monitored during HIV prevention or treatment will decrease.

The market price for a creatinine test at a major private laboratory in Eswatini’s capital Mbabane was between $3.32 and $4.01 in the years 2017–19. Public primary care clinics, which provide PrEP and other HIV services in Eswatini, however, use government referral laboratories at no charge but a cost to the public health system.

This micro-costing study assessed the costs of creatinine testing for blood samples that were collected in public primary care clinics and analyzed in the government referral laboratory of the Hhohho region in Eswatini during an early phase of PrEP expansion. The average cost of creatinine testing as well as the cost of performing additional creatinine tests for PrEP were estimated. Knowledge of these costs can inform cost and cost-effectiveness analyses of PrEP and other health services that involve creatinine testing or help plan laboratory budgets and investments.

## Methods

### Study Setting

Eswatini is a landlocked country bordering South Africa and Mozambique. Its resident population was 1.1 million people, of which 29% lived in the Hhohho region, according to the last population census in 2017 [9]. The whole country is heavily burdened by HIV with little geographic variation of the HIV prevalence across regions. Prior to the start of a PrEP demonstration project in the Hhohho region, the national HIV prevalence among adults aged 15 years and older was 27%. The HIV prevalence peaked in 35–39-year-old women (54%) and 45–49-year-old men (49%) [10].

Initially, PrEP was only available in Eswatini through demonstration projects. In addition to the PrEP demonstration project in the Hhohho region, in which this study was nested, there were two PrEP demonstration projects in the Manzini and Shiselweni regions. A nationwide scale-up of PrEP that began in 2019 was decided in 2018 [11]. According to our information, the nationwide PrEP program targets include reaching almost 25,000 people at its peak in the financial year 2022.

The Hhohho region is in the northwestern part of Eswatini and includes the capital, Mbabane. To our knowledge, there were six private laboratories in the region in early 2020 and one government referral laboratory. Public primary care clinics in Hhohho and the other regions of Eswatini usually conduct rapid tests on-site and send blood samples for clinical chemistry to the regional government referral laboratory.

The costs of blood sample collection in a public primary care clinic and serum creatinine testing in the government referral laboratory of the Hhohho region were assessed during the PrEP demonstration project in the region in 2017–19. During the 18 months demonstration project, PrEP was provided to over 500 PrEP clients in six public primary care clinics which may have served about 20% of the Hhohho population in 2016/17 [6, 9]. To monitor serum creatinine levels, blood samples should have been regularly taken from PrEP clients during clinic visits—at PrEP initiation and then 6-monthly for clients continuing PrEP according the demonstration project guidelines [12, 13]. Collected blood samples were sent to the Hhohho government referral laboratory. The laboratory determined serum creatinine levels with a Beckman Coulter/Olympus AU480 chemistry analyzer based on a modified Jaffe method.

### Costing Approach and Cost Classification

An activity-based costing approach [14] was chosen in which we identified activities related to serum creatinine testing in the clinic and laboratory. For each activity, we identified and quantified the resources required to perform one creatinine test. Average resource costs per creatinine test were calculated by multiplying the resource use per test and the resource unit cost, that is, the cost of one unit of a resource. The average cost of creatinine testing was calculated by summing-up the average resource costs over resources required for creatinine testing.

To account for administration, machine maintenance, transportation, waste disposal, and other potentially excluded costs, like the license fees for the laboratory information system, a 10% overhead was added to the total resource cost. Furthermore, 5% cumulative interest per year over an assumed 10-year lifetime was added to costs of laboratory equipment to account for capital costs. Capital costs could represent the cost of a loan for purchasing laboratory equipment or an opportunity cost of being unable to use capital invested in laboratory equipment for other purposes.

Costs that changed with a change in testing activity were classified as variable costs if it seemed plausible that a resource was fully used or could have been used elsewhere (e.g., blood sample collection tubes, creatinine testing reagents, or personnel time). Costs that were—to some degree—independent of testing activity were classified as semi-fixed costs (e.g., daily machine maintenance or the purchase of laboratory equipment).

### Data Sources

Creatinine testing resources and required quantities were estimated for the locally implemented creatinine testing procedure by an Eswatini-based laboratory technical advisor of the Clinton Health Access Initiative (RN) in deliberations with the laboratory head of the Mbabane Government Hospital (SD). Prices for valuing the inputs in creatinine testing stem from 2017/18 biannual government procurement tenders and annual salary schemes. Prices for machinery in the laboratory (chemical analyzer, centrifuge), electricity, rent, and printing were own estimates (SK) based on an internet search. Test numbers were extracted from the laboratory information system. For laboratory machines, that is, the AU480 chemistry analyzer and a Beckman Coulter Allegra X-22 benchtop centrifuge, a maximum annual test capacity was estimated based on technical information for the throughput and an operating time of 40 h per week (SK). Costs were recorded in Eswatini Lilangeni (SZL) and converted to United States dollar ($) using the official average exchange rate for 2018 of $1 = SZL13.234 [15].

### Costing Assumptions and Sensitivity Analysis

Personnel costs were assumed to be variable costs and estimated as the mean of the minimum and maximum salary level of a health worker cadre. Salaries per minute were calculated based on 220 workdays per year and eight workhours per day. Utilization rates of laboratory equipment were estimated, assuming all samples processed in the chemistry analyzer were also centrifuged. Costs for machinery were based on purchasing prices for comparable used machinery outside of Eswatini. The considered 10% overhead cost and 5% interest rate were ad hoc assumptions. In addition, our costing assumed that all tests could be performed at the same resource costs. To assess how changes in the measured or assumed costs of the largest cost contributors affect creatinine testing costs, univariate sensitivity analyses were conducted.

## Results

### Resource Use and Costs

We studied creatinine testing as a process that started with drawing a blood sample in a public primary care clinic and ended with the printing and delivery of the sample analysis results from the referral laboratory to the clinic. We identified the following activities in this process:

1. Drawing blood in the clinic,
2. Transporting blood samples from clinics to the laboratory,
3. Daily preparation and re-calibration of the chemistry analyzer for creatinine testing,
4. Centrifugation of blood samples,
5. Further processing of small-volume blood samples,
6. Analyzing centrifuged blood samples and documenting results, and
7. Transporting printed results from the laboratory to clinics.

The estimated types and amounts of resources used in these activities as well as the corresponding cost for one unit of each resource are summarized in Table 1.

**Table 1:** Activity-based identification of resources, resource use and costs, and unit costs for creatinine testing in the Eswatini public health care system

Activity	Resource	Resource type	Cost type	Resource use	Cost per resource unit ($)	Cost per creatinine test ($)
Blood draw	Phlebotomist (per minute)	Personnel	Variable	5 min. per blood draw	0.063	0.32
	Gloves	Consumable	Variable	2 per blood draw	0.047	0.094
	Alcohol swab	Consumable	Variable	1 per blood draw	0.038	0.038
	Multi-sample needle	Consumable	Variable	1 per blood draw	0.053	0.053
	Red-top tubes (4–5 ml)	Consumable	Variable	1 per blood draw	0.067	0.067
	Sharp disposal bin (7 l)	Consumable	Variable	1 per 140 blood draws	5.66	0.04
	Strapping tape	Consumable	Variable	1 per 100 blood draws	0.71	0.0071
	Gauze swab	Consumable	Variable	1 per blood draw	0.016	0.016
Daily preparation of chemistry analyzer for creatinine testing	Lab technician (per minute)	Personnel	Semi-fixed	30 min. per day	0.11	0.0013
	Control serum 1 (20 × 5 ml)	Consumable	Semi-fixed	3 per year	19.09	0.00045
	Control serum 2 (20 × 5 ml)	Consumable	Semi-fixed	3 per year	21.21	0.00050
Centrifugation of blood samples	Lab technician (per minute)	Personnel	Variable	2 min. per creatinine test	0.11	0.21
	Gloves	Consumable	Variable	2 per batch of 20 tests	0.047	0.0047
	Electricity per workday	Consumable	Semi-fixed	1 per day	0.68	0.00027
	Benchtop centrifuge (Allegra X-22)	Lab equipment	Semi-fixed	1 per 10 years	5000	0.00075
	Micropipette	Lab equipment	Semi-fixed	1 per 10 years	231	0.000035
Blood sample analysis and documentation	Lab technician (per minute)	Personnel	Variable	8 min. per test	0.11	0.85
	Gloves	Consumable	Variable	2 per batch of 20 tests	0.047	0.0047
	Wash solution (4 × 2 l)	Consumable	Variable	1 per 5000 tests	37.05	0.0074
	Creatinine reagents (OSR6178)	Consumable	Variable	1 per 200 creatinine tests	11.1	0.055
	Printout of results	Consumable	Variable	1 page per 20 tests	0.038	0.0019
	Electricity per workday	Consumable	Semi-fixed	1 per day	4.34	0.0017
	50 micron pre-filter^*^	Consumable	Semi-fixed	2 per year	107	0.00032
	0.05 µm ultra-filter	Consumable	Semi-fixed	1 per year	838	0.0013
	0.2 µm capsule filter	Consumable	Semi-fixed	1 per year	961	0.0015
	Chemistry analyzer (AU480)	Lab equipment	Semi-fixed	1 per 10 years	70,000	0.011
Other	Overhead clinic	Other	Variable	10% of clinic resource costs		0.063
	Overhead lab	Other	Variable	10% of lab resource costs		0.12
	Capital cost	Other	Semi-fixed	3% of equipment cost over 10 years	47,312	0.0071
	Imputed rent	Other	Semi-fixed	1 per year	907	0.0014
Processing of small volume samples^*^	Lab technician (per minute)	Personnel	Variable	1 per small volume sample	0.11	0.11
	Transparent sample cup	Consumable	Variable	1 per small volume sample	0.53	0.53
	Disposable pipette^*,^^✝^	Consumable	Variable	1 per small volume sample	0.022	0.022
	Pipette tips (5–200 µl)	Consumable	Variable	1 per small volume sample	0.0047	0.0047

*Not included in base case

^✝^Disposable pipettes are a more costly substitute for a re-usable micropipette with disposable tips. *Data sources:* Own estimation of resources and use. 2017/18 Government of Eswatini procurement and salary information

Based on mean salaries, 220 workdays per year, and eight workhours per day, we estimated personnel costs per minute of ¢6 for a phlebotomist and ¢11 for a lab technician. The costs of consumable resources ranged from ¢47 for a pipette tip to $961 for a water filter. The estimated costs of lab equipment were $231 for a re-usable pipette, $5000 for a benchtop centrifuge, and $70,000 for the chemistry analyzer. Costs for space in the laboratory buildings were considered by assuming an imputed rent of $907 per year. Transportation costs were approximated together with costs for administration, machine maintenance, and other potentially excluded costs as an overhead of 10% on the explicitly assessed resource costs. The capital costs of 5% per year over 10 years for laboratory equipment added $47,312 (+ 63%) on top of the estimated equipment costs of $75,231 (100%) for the AU480 chemistry analyzer, the Allegra X-22 benchtop centrifuge, and a re-usable micropipette.

### Costs Per Creatinine Test

**Fig. 1:**
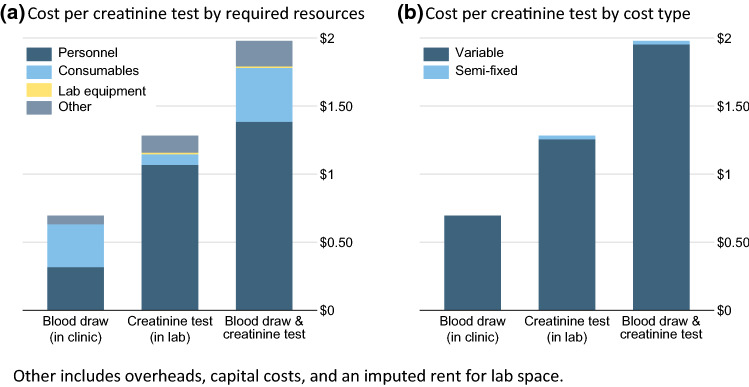
The cost of serum creatinine testing in the Eswatini public health care system

The resource costs per creatinine test ranged from ¢0.027 for the electricity to power the chemistry analyzer and centrifuge to ¢85 for lab technician time for the blood sample analysis (Table 1). The total resource costs per creatinine test are presented in Fig. 1 and Table 2. Obtaining a blood sample in a public primary care clinic in Eswatini was estimated to cost ¢70. Performing one additional serum creatinine test in the government laboratory of the Hhohho region was estimated to cost US$ 1.28 for a blood sample of sufficient volume (red-top tube filled with 2–4 ml of blood or more during sample collection). The average total cost of creatinine testing was estimated at US$ 1.98 (100%), including $1.39 (70%) personnel costs, ¢39 (20%) consumables costs, ¢1.1 (0.6%) lab equipment costs, and ¢19 (9.5%) for overheads, capital costs and rent. Variable costs (listed in Table 1) were associated with $1.95 (99%) and semi-fixed costs with ¢2.6 (1.3%) of the cost of a creatinine test.

**Table 2:** The cost of serum creatinine testing in the Eswatini public health care system

Location, activity, and resource type	Cost per test ($ (%))	Variable cost per test ($ (%))	Semi-fixed cost per test ($ (%))
Clinic			
Blood draw	0.63 (32)	0.63 (32)	
– Personnel	0.32 (16)	0.32 (16)	
– Consumables	0.32 (16)	0.32 (16)	
Overhead	0.063 (3.2)	0.063 (3.2)	
Total	0.7 (35)	0.7 (35)	
Lab			
Calibration of chemistry analyzer	0.0022 (0.1)		0.0022 (0.1)
– Personnel	0.0013 (0.1)		0.0013 (0.1)
– Consumables	0.001 (0.05)		0.001 (0.05)
Centrifugation of blood samples	0.22 (11)	0.22 (11)	0.001 (0.1)
– Personnel	0.21 (11)	0.21 (11)	0 (0)
– Consumables	0.005 (0.3)	0.0047 (0.2)	0.00027 (0.01)
– Lab equipment	0.00075 (0.04)		0.00075 (0.04)
Blood sample analysis and documentation	0.94 (47)	0.92 (47)	0.014 (0.7)
– Personnel	0.85 (43)	0.85 (43)	
– Consumables	0.073 (3.7)	0.069 (3.5)	0.0033 (0.2)
– Lab equipment	0.011 (0.5)		0.011 (0.5)
Other	0.12 (6.3)	0.12 (5.9)	0.0085 (0.4)
– Overhead	0.12 (5.9)	0.12 (5.9)	
– Capital cost	0.0071 (0.4)		0.0071 (0.4)
– Imputed rent	0.0014 (0.1)		0.0014 (0.1)
Total	1.28 (65)	1.26 (64)	0.026 (1.3)
Clinic and lab			
– Personnel	1.39 (70)	1.38 (70)	0.0013 (0.06)
– Consumables	0.39 (20)	0.39 (20)	0.0045 (0.2)
– Lab equipment	0.011 (0.6)		0.011 (0.6)
– Other	0.19 (9.5)	0.18 (9.1)	0.0085 (0.4)
Total	1.98 (100)	1.95 (99)	0.026 (1.3)

() = % of the total cost of $1.98 per creatinine test in clinic and lab

Looking at the costs of blood sample collection and blood sample analysis separately, the estimated 5 minutes time of a phlebotomist in the clinic for drawing a blood sample contributed ¢32 (16%) to the creatinine testing cost. Consumables used when drawing a blood sample were estimated to cost another ¢32 (16%). In the regional government laboratory, the estimated 10 minutes lab technician time for performing a creatinine test contributed $1.07 (54%) to the creatinine testing cost. Consumables required for determining the creatinine level in the laboratory (i.e., creatinine reagents and wash solution) were estimated to cost ¢7.9 (4.0%) for each creatinine test. Lab equipment cost contributed ¢1.1 (0.6%) under the assumption that the estimated 2018 utilization rates of 79% for the chemistry analyzer and 50% for the benchtop centrifuge were maintained over an equipment lifetime of 10 years.

### Univariate Sensitivity Analysis

**Fig. 2:**
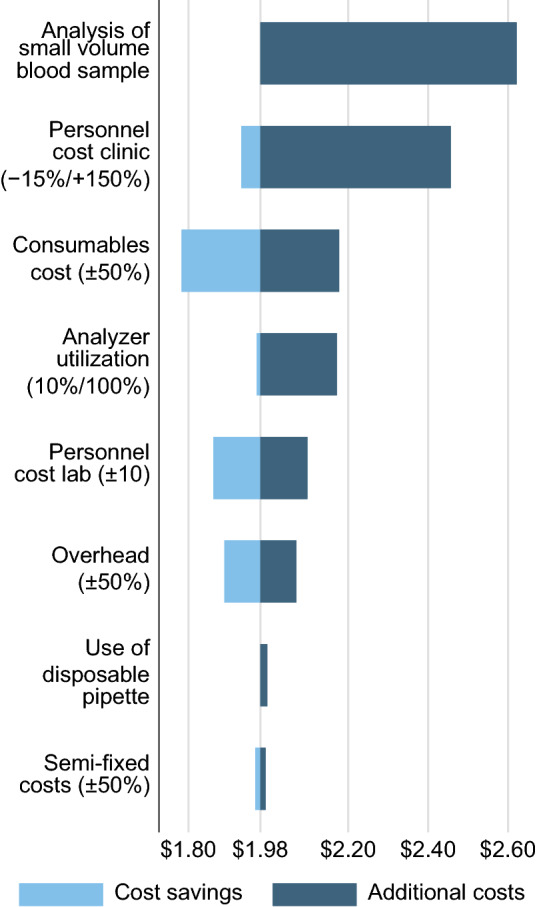
Univariate sensitivity analysis of the costs of serum creatinine testing in the Eswatini public health care system

The univariate sensitivity analysis (Fig. 2) indicated that the additional processing required for a small blood sample (red-top tube filled with less than 2–4 ml of blood) in the laboratory increased the cost per creatinine test to $2.62 (+ 32% added to the creatinine testing cost for a non-small blood sample). The sample cup into which a small volume blood sample needs to be transferred for analysis caused the highest per-test consumable cost of ¢53. Additional lab technician time (¢11) and the need to pipette blood serum (¢47) caused the remaining portion of the additional costs for small sample analysis. Furthermore, while a small cost contributor in absolute terms, using a disposable pipette was more costly per test than using a re-usable pipette with disposable tips.

Another factor that affects the estimated creatinine testing cost is the salary of the health care worker collecting the blood sample. Our base case used the mean salary level of a phlebotomist. At some public primary care clinics, a nurse and not a phlebotomist may perform the blood collection. According to the 2018 salary scheme and depending upon the qualification level, the average salaries for nurses were about 100% to 200% higher than those used in the base case analysis. A nurse taking a blood sample could, therefore, increase the costs of a creatinine test to the health system to $2.46 (+ 23%) or more. On the other hand, if less time than 5 minutes is needed for taking a blood sample or if a blood sample is taken for more than one test, then costs of creatinine testing decrease below our base case estimate. For lab personnel, we varied costs in a narrower range than for clinic personnel within the sensitivity analysis because lab technician salary levels in 2018 were within ± 6% of the base case. As consumables caused ¢39 (20%) of the estimated creatinine testing cost of $1.98, fluctuations in their cost can impact the estimated creatinine testing relatively strongly. Fluctuations in the semi-fixed cost, in turn, showed a minor impact on the testing cost as all semi-fixed costs were allocated to a relatively large number of tests over the lab equipment lifetime, which was 10-times the number of tests performed in the Hhohho referral laboratory in 2018.

### Utilization of Laboratory Equipment

A total number of 662,453 blood tests, including 126,920 (19%) creatinine tests, were performed in the Hhohho government referral laboratory in 2018 according to the laboratory information system. This test volume corresponds to estimated utilization rates of 79% for the chemistry analyzer and 50% for the benchtop centrifuge if all blood samples are centrifuged. The cost of $1.98 per creatinine test at this laboratory utilization level is close to the estimated $1.97 minimum cost at 100% utilization rate of the chemistry analyzer. If the laboratory utilization decreased to 200,000 blood tests, including 20,000 creatinine tests, per machine, for instance, due to a doubling the laboratory capacity combined with demand decrease, the estimated cost per creatinine test would increase to $2.05 (+ 3.4%).

Table 3 presents a detailed sensitivity analysis of the cost per creatinine test for a variation in test numbers. Changes to both the number of blood tests and the number of creatinine tests affect creatinine testing costs. The number of blood tests affects the share of the laboratory fixed costs assigned to the average cost of any performed test, whereas the number of creatinine tests affects the share of the creatinine-specific fixed costs (i.e., the daily preparation and re-calibration of the chemistry analyzer for creatinine testing) assigned to each creatinine test performed. We estimated that the average creatinine testing cost in the public health care system could become lower than a 2018 market price of $3.65 when the annual test throughput of the laboratory surpasses 20,000 blood tests, including 2000 creatinine tests, per year over 10 years.

**Table 3:** Sensitivity of the cost of serum creatinine testing with respect to lab equipment utilization

Cost per creatinine test ($)		Number of creatinine tests per year												Utilization (%)	
		100	200	500	1000	2000	5000	10,000	20,000	50,000	100,000	126,920	200,000	AU480	Allegra X-22
Total number of tests per year	1000	20.94	20.15	19.67	19.51									0.1	0.1
	2000	12.24	11.44	10.97	10.81	10.73								0.2	0.1
	5000	7.02	6.22	5.75	5.59	5.51	5.46							0.6	0.4
	10,000	5.28	4.48	4.01	3.85	3.77	3.72	3.71						1.2	0.7
	20,000	4.41	3.61	3.14	2.98	2.90	2.85	2.84	2.83					2.4	1.5
	50,000	3.88	3.09	2.62	2.46	2.38	2.33	2.32	2.31	2.30				6.0	3.7
	100,000	3.71	2.92	2.44	2.28	2.20	2.16	2.14	2.13	2.13	2.13	2.13		12	7.5
	200,000	3.62	2.83	2.36	2.20	2.12	2.07	2.05	2.05	2.04	2.04	2.04	2.04	24	15
	331,227	3.59	2.80	2.32	2.16	2.08	2.04	2.02	2.01	2.01	2.01	2.01	2.00	40	25
	662,453	3.56	2.77	2.29	2.14	2.06	2.01	1.99	1.99	1.98	1.98	1.99	1.99	79	50
	834,286	3.56	2.76	2.29	2.13	2.05	2.00	1.99	1.98	1.98	1.97	1.98	1.98	100	63

We estimated maximum capacities of 834,286 tests per year for the AU480 chemistry analyzer and 1,334,858 tests per year for the Allegra X-22 benchtop centrifuge

### Costs of Additional Creatinine Tests for PrEP Care

PrEP guidelines recommended creatinine testing in PrEP clients every 6 months during the demonstration project. Combining this recommended test frequencies with a targeted maximum of 25,000 clients in the nationwide program, while assuming that 30% of the PrEP clients would live in the Hhohho region, suggests that the annual demand for creatinine testing in the region’s referral laboratory could increase by up to 15,000 (+ 12%) in comparison to 126,920 creatinine tests in the study year 2018. Such an increase in creatinine testing could be feasible within the estimated existing laboratory capacity. An increase in the test volume by 15,000 creatinine tests would result in similar costs of $1.98 per creatinine test or even a small average cost decrease if the total annual test number remained at 662,453 or higher. If the laboratory expanded at the given high utilization level and, for instance, doubled its capacity, then the utilization rate would decrease from 79% to 40% per chemistry analyzer and the creatinine testing cost would increase to $2.01 according to our sensitivity analysis (Table 3).

## Discussion

### Summary of Findings

As access to PrEP is expanding in several sub-Saharan African countries [16], there is considerable interest in the costs and cost-effectiveness of PrEP approaches [17, 18]. We estimated the cost of creatinine testing, which is frequently performed for HIV treatment and prevention as well as other chronic disease care, in a recent and natural context. Specifically, we conducted activity-based costing to estimate the average cost per serum creatinine test performed in the Eswatini public health care system. Creatinine testing costs within the given laboratory capacity and under a capacity expansion were estimated. Both were of interest because Eswatini is presently expanding PrEP provision to everyone at high risk of acquiring an HIV infection [11]. This expansion of PrEP care involves additional serum creatinine testing that—up to a certain extent—can be performed within the existing health infrastructure.

Collecting a blood sample in a public primary care clinic (¢70 per blood sample) and performing a creatinine test in the Hhohho government referral laboratory ($1.28 per creatinine test) was estimated to be feasible at an average cost of $1.98 in 2018 if the blood draw was performed by a phlebotomist. Related to a large number of blood tests performed in the referral laboratory, $1.94 (98%) of the total average creatinine testing cost were variable costs and only ¢4 (2%) were semi-fixed costs. The largest share of the total average creatinine testing cost was due to personnel costs ($1.39 or 70%), followed by consumables costs (¢39 or 20%), laboratory equipment costs (¢1.1 or 0.6%), and other costs (¢19 or 9.5%). Other costs included overhead costs (¢18 or 9.1%), capital costs (¢0.71 or 0.4%), and imputed rent (¢0.14 or 0.1%).

A univariate sensitivity analysis indicated two variations in resource use and costs that affect the estimated creatinine testing cost by more than ± ¢40 (± 20%). Firstly, the estimated cost per creatinine test increases from $1.98 to $2.62 (+ 32%) if the laboratory receives a small volume blood sample that requires transfer from a red-top tube to a small sample cup before analysis. Secondly, if a nurse (single or higher qualified) rather than a phlebotomist takes a blood sample, then a substantially higher blood sample collection cost could increase the cost of creatinine testing to $2.46 (+ 24%) or more. The creatinine testing cost was relatively robust with respect to a change in the laboratory utilization when certain minimum test numbers to cover the laboratory’s semi-fixed cost can be maintained. Performing more than 20,000 blood tests, including at least 200 creatinine tests, per year over 10 years appeared sufficient for performing creatinine tests at a cost below the 2018 market price of $3.65 of a large private laboratory in Eswatini. While these test numbers appear relatively low compared to the observed test numbers in 2018 (> 600,000 blood tests, > 100,000 creatinine tests in the laboratory), these numbers would have to be sustainably maintained over several years. Furthermore, the demand for tests would have to be reasonably steady over time such that the allocation of an equal share of the semi-fixed costs to each test, as performed in this costing study, is a plausible approximation to the testing reality.

### Other Creatinine Test Cost Data

A study of 73 private and 5 non-private, randomly selected laboratories in Kampala, Uganda, reported 2017 market prices between $1.47 and $11.76 for a creatinine test in prepared study samples [19]. A cost analysis conducted in 2017 in South Africa reported costs of $3.41 per creatinine test (¢78 personnel costs, ¢42 clinic consumables costs, and $2.21 laboratory and transport costs) [20]. Past cost and cost-effectiveness analyses of PrEP in sub‐Saharan Africa have used creatinine testing costs of 2016 $2.16 [21], 2010 $3.44 to $6.87 [22], 2014–19 $4 [23], 2012 $4.17 [24], approximately 2017 $4.50 [25], and 2012 $8.50 [26] in their assessments of PrEP costs for South Africa, Nigeria and Uganda. Latter creatinine testing costs were derived from government commodity tender prices, budgets, invoices, or the national health laboratory service with sparse or no documentation about the cost components included in the analyses [21–26]. The newer findings of the micro-costing study at hand suggest that creatinine testing in a sub‐Saharan Africa health system in 2018 was feasible at a lower cost than assumed in past PrEP cost and cost-effectiveness analyses.

Private laboratories also offer lab services—in Eswatini mainly in the Hhohho and Manzini regions. We researched market prices for a creatinine test at a major private laboratory in the Eswatini capital Mbabane and found prices, including the blood draw, of US$ 3.32, US$ 3.65 and US$ 4.01 for the years 2017, 2018 and 2019, respectively. Compared to these market prices, we estimated a lower cost for creatinine testing in the Eswatini public health care system.

For creatinine point-of-care testing, a cost of US$ 3.40 per test strip was reported in the context of a health study in Haiti in 2015/16 [27]. Cost analyses for point-of-care creatinine testing in South Africa in 2017, in turn, reported creatinine testing costs of $9 and $10 for point-of-care testing [20, 25], of which $8 were contributed by the test strip in one study [20]. These reported, higher costs for a point-of-care creatinine testing suggest that sending blood samples to a high-throughput laboratory for creatinine testing is more cost-effective than point-of-care creatinine testing, unless point-of-care creatinine testing costs fall. Conversely, point-of-care creatinine testing could be cost-effective at a cost above our cost estimate of $1.98 per creatinine test in settings with high sample transport costs or higher laboratory costs than in a high-throughput laboratory.

### Practical Implications

The findings of this study can inform the planning, budgeting, and evaluation of PrEP care and other health programs that involve creatinine testing. The estimated costs of taking a blood sample and performing a creatinine test in a referral laboratory may be used for cost and cost-effectiveness analyses of PrEP in Eswatini and comparable settings. The study of variable and semi-fixed costs suggested that the average testing costs were predominantly caused by variable costs in the setting of a laboratory with high throughput. The dominance of the variable costs implies that capacity expansion could be a secondary concern from a health care payer perspective when a stable and long-term demand is expected.

Further, we identified cost contributors that appear amendable to modification. We estimated a relatively high additional cost for testing the serum creatinine level in a small volume blood sample. The cost to prepare a small volume sample for analysis increased the cost of a creatinine test from $1.98 to $2.62. This cost increase was largely caused by a small sample cup purchased at a unit price of ¢53 in 2017/18. For comparison, a red-top tube used for blood sample collection, from which a blood sample of sufficient volume can be directly analyzed, cost ¢7. Investigating the reasons for the relatively high purchasing price of small volume cups in 2017/18 or the frequency at which small samples arrived in the laboratory was beyond the scope of this study. The finding of a large cost difference between regular and small volume blood samples in our data snapshot, however, could point to an area for process improvement. Future enquiry could ask questions, like: How might we procure less costly small sample cups, or how might we increase the number of sufficiently filled sample collections tubes? Another, potentially avoidable cost increase from $2.62 to $2.64 was estimated when a disposable pipette instead of a re-usable pipette with a disposable tip was used for small sample processing. While the use of a disposable pipette results in a relatively small cost increase, it might be avoidable easily if a re-usable pipette is available and used.

### Strengths and Limitations

The activity-based costing approach of this study facilitated a comprehensive bottom-up assessment of the financial costs and capital costs of creatinine testing (compare, e.g., [28, 29]). Resource costs were based on actual government procurement prices and salary information. Limitations of the study include the pragmatic, expert-based estimation of resource use. Estimated resource use may differ from actual practice. Further, this costing study assumed that laboratory equipment is purchased, whereas the referral laboratory leased rather than purchased some equipment. Also, some resource costs were assumed or derived from an internet search for purchasing prices of comparable used machinery outside of Eswatini.

Regarding the transferability of our findings to other contexts, a creatinine test may be performed in a blood sample drawn also for other blood tests, reducing the costs associated with a creatinine test. Finally, the prices of the resources used in creatinine testing might have changed since 2017/18 as indicated by consumer prices increases of 6.2%, 4.8% and 2.6% in 2017, 2018 and 2019, respectively, in Eswatini [30]. To mitigate these limitations and assess the robustness of the estimated creatinine testing costs, changes in the costs of the largest cost contributors were simulated in a sensitivity analysis.

## Conclusions

Obtaining a blood sample and performing one creatinine test was estimated to have cost $1.98 in the Eswatini public health care system in 2018. Simulating different utilization of the laboratory indicated that the semi-fixed costs of the laboratory (e.g., costs for equipment or daily calibration of the chemistry analyzer) have a minor impact on testing costs when the laboratory maintains certain minimum test numbers. Our findings indicate a lower creatinine testing cost than used in past cost and cost-effectiveness analyses of PrEP and other HIV services or reported for point-of-care creatinine testing. Further, our findings suggest that investment in lab equipment for clinical chemistry, like creatinine testing, imposes a relatively small additional cost on each performed test in a high-throughput laboratory.

## Data Availability

The data and code that support the findings of this study are openly available in heiDATA at 10.11588/data/KXTQTZ [31].
